# A differential diagnosis between uterine leiomyoma and leiomyosarcoma using transcriptome analysis

**DOI:** 10.1186/s12885-023-11394-0

**Published:** 2023-12-08

**Authors:** Kidong Kim, Sarah Kim, TaeJin Ahn, Hyojin Kim, So-Jin Shin, Chel Hun Choi, Sungmin Park, Yong-Beom Kim, Jae Hong No, Dong Hoon Suh

**Affiliations:** 1 Department of Obstetrics and Gynecology, Seoul National University Bundang Hospital, Seongnam, Republic of Korea; 2 Department of Life Science, Handong Global University, Pohang, Republic of Korea; 3 Department of Pathology, Seoul National University Bundang Hospital, Seongnam, Republic of Korea; 4 Department of Gynecology and Obstetrics, School of Medicine, Keimyung University, Daegu, Republic of Korea; 5 Department of Obstetrics and Gynecology, Sungkyunkwan University School of Medicine, Seoul, Republic of Korea

## Abstract

**Background:**

The objective of this study was to estimate the accuracy of transcriptome-based classifier in differential diagnosis of uterine leiomyoma and leiomyosarcoma. We manually selected 114 normal uterine tissue and 31 leiomyosarcoma samples from publicly available transcriptome data in UCSC Xena as training/validation sets. We developed pre-processing procedure and gene selection method to sensitively find genes of larger variance in leiomyosarcoma than normal uterine tissues. Through our method, 17 genes were selected to build transcriptome-based classifier. The prediction accuracies of deep feedforward neural network (DNN), support vector machine (SVM), random forest (RF), and gradient boosting (GB) models were examined. We interpret the biological functionality of selected genes via network-based analysis using GeneMANIA. To validate the performance of trained model, we additionally collected 35 clinical samples of leiomyosarcoma and leiomyoma as a test set (18 + 17 as 1st and 2nd test sets).

**Results:**

We discovered genes expressed in a highly variable way in leiomyosarcoma while these genes are expressed in a conserved way in normal uterine samples. These genes were mainly associated with DNA replication. As gene selection and model training were made in leiomyosarcoma and uterine normal tissue, proving discriminant of ability between leiomyosarcoma and leiomyoma is necessary. Thus, further validation of trained model was conducted in newly collected clinical samples of leiomyosarcoma and leiomyoma. The DNN classifier performed sensitivity 0.88, 0.77 (8/9, 7/9) while the specificity 1.0 (8/8, 8/8) in two test data set supporting that the selected genes in conjunction with DNN classifier are well discriminating the difference between leiomyosarcoma and leiomyoma in clinical sample.

**Conclusion:**

The transcriptome-based classifier accurately distinguished uterine leiomyosarcoma from leiomyoma. Our method can be helpful in clinical practice through the biopsy of sample in advance of surgery. Identification of leiomyosarcoma let the doctor avoid of laparoscopic surgery, thus it minimizes un-wanted tumor spread.

**Supplementary Information:**

The online version contains supplementary material available at 10.1186/s12885-023-11394-0.

**Pre´cis**.

A new transcriptome-based classifier was able to accurately distinguish uterine leiomyoma from leiomyosarcoma using data from preoperative biopsy specimens.

## Introduction

Uterine leiomyoma is a common, benign smooth muscle tumor arising from the uterus. In a cross-sectional study, the estimated cumulative incidence of leiomyomas by the age of 50 years was over 70% [1]. Uterine leiomyosarcoma is a rare malignancy arising from smooth muscle cells of the uterus and is usually diagnosed postoperatively. A study reported that the risk of occult uterine leiomyosarcoma is 17.3 per 10,000 laparoscopic supracervical hysterectomy or myomectomy [2].

Preoperative differential diagnosis between uterine leiomyoma and leiomyosarcoma is important because it affects treatment and prognosis. For example, suspected malignancy is one of the indications for hysterectomy in women with uterine leiomyoma [3]. Consequently, many asymptomatic women with uterine leiomyoma undergo a hysterectomy because they worry about the risk of uterine leiomyosarcoma. Additionally, preoperatively undiagnosed uterine leiomyosarcoma can result in reduced survival, especially in the case of unintentional morcellation of uterine leiomyosarcoma [4].

However, preoperative differential diagnosis between uterine leiomyoma and leiomyosarcoma is difficult. Differential diagnosis using sonography is inaccurate due to its limited soft-tissue characterization [5]. Even magnetic resonance imaging (MRI) cannot reliably distinguish uterine leiomyoma and leiomyosarcoma [6]. Although advanced imaging techniques such as multiparametric MRI and positron emission tomography were promising, they have limitations [6]. Similarly, preoperative tumor biopsy reported an overlap in histopathologic score between uterine leiomyoma and leiomyosarcoma [3]. It was suggested that the small size of biopsy specimens can result in mis-diagnosis because standard histopathological evaluation of uterine leiomyosarcoma requires multi-section examination [3].

We hypothesized that transcriptome analysis would be useful in differential diagnosis between uterine leiomyoma and leiomyosarcoma. The differential diagnosis via transcriptome analysis of preoperative biopsy specimens may lead to optimization of treatment plan in women with uterine leiomyoma or leiomyosarcoma. The objective of this study was to estimate the accuracy of transcriptome-based classifiers in the differential diagnosis of uterine leiomyoma and leiomyosarcoma.

## Methods

### Data set

We used 19,131 transcriptomes from UCSC Xena which provides pre-processed integrated data of the Cancer Genome Atlas (TCGA), tumor alterations relevant for genomic driven therapy (TARGET), and Genotype Tissue Expression (GTEx) databases in fragment per kilobase of transcript per million mapped reads (FPKM) values.

We classified the 19,131 transcriptomes by their tissue of origin. There were 8,167 transcriptomes from normal tissue, and for 114 samples, the primary sites were uterus (n = 78), cervix (n = 3), cervix uteri (n = 10), or endometrium (n = 23). We used these 114 samples for classifier generation, using 92 samples for gene selection/training and 22 samples for validation (Table 1).

**Table 1:** The number of samples in the training, validation, and test sets

	Classifier generation		Classifier evaluation		
	TCGA TARGET GTEx		Clinical validation samples		
	Training	Validation	Test − 1st	Test − 2nd	Subtotal
Leiomyosarcoma	25	6	9	9	49
Normal tissue (uterus, cervix, cervix uteri, endometrium)	92	22	0	0	114
Leiomyoma	0	0	9	8	17
Subtotal	117	28	18	17	180

We manually selected 258 sarcoma samples from the 19,131 transcriptomic data where their annotation showed primary tumor. We chose 31 sarcoma samples from uterine sites including corpus uteri (n = 4) or uterus (n = 27). The primary diagnoses of the 31 samples were leiomyosarcoma (n = 29) or myxoid leiomyosarcoma (n = 2). In this study, we considered the 31 uterine-origin sarcoma samples as leiomyosarcoma and used these samples for training (n = 25) / validation (n = 6) of the prediction model.

We manually selected 114 normal uterine tissue and 31 leiomyosarcoma samples from publicly available transcriptome data for this study (Table 1).

We collected clinical samples of leiomyoma and leiomyosarcoma cases. Samples were collected from different research sites and sequenced in two different batches. For the 1st sequenced data, 22 samples from a tissue repository were collected. After excluding 4 samples with poor RNA quality or failed library construction, 18 samples (9 uterine leiomyoma and 9 uterine leiomyosarcoma) with FPKM values of 23,043 genes were categorized as the 1st test set. For the 2nd sequenced data, 27 samples from two tissue repositories were collected. After excluding 10 samples with poor RNA quality or failed library construction, 17 samples (8 uterine leiomyoma and 9 uterine leiomyosarcoma) with FPKM values of 23,043 genes were categorized as the 2nd test set (Table 1).

The training/validation set was used to generate classifiers, and the 1st and 2nd test sets were used to measure the performance of the classifiers. Test sets were never used during training the prediction classifiers, and the best classifier was chosen based solely on the 1st test set. Thus, the 2nd test set can be considered as an independent validation set.

### RNA sequencing in test sets

The libraries were prepared for 151 bp paired-end sequencing using the TruSeq Stranded mRNA Sample Preparation Kit (Illumina, San Diego, CA, USA). mRNA molecules were purified and fragmented from 1 µg of total RNA using oligo (dT) magnetic beads. The fragmented mRNAs were synthesized as single-stranded cDNAs through random hexamer priming. By applying this as a template for synthesis of the second strand, double-stranded cDNA was prepared. After sequential process of end repair, A-tailing, and adapter ligation, cDNA libraries were amplified with PCR (polymerase chain reaction). Quality of these cDNA libraries was evaluated using the Agilent 2100 BioAnalyzer (Agilent, Santa Clara, CA, USA). They were quantified with the KAPA library quantification kit (Kapa Biosystems, Wilmington, MA, USA) according to the manufacturer’s library quantification protocol. Following cluster amplification of denatured templates, sequencing was progressed as paired-end (2 × 151 bp) using Illumina NovaSeq6000 (Illumina).

The Phred quality score of 30 indicates a probability of 1 in 1000 for an incorrect base call. To ensure high confidence in the sequencing data, adapter sequences and ends of reads with a Phred quality score less than 30 were trimmed, and simultaneously, reads shorter than 50 bp were removed using cutadapt v.2.8 [7].

Filtered reads were mapped to the human reference genome (GRCh38 from ENSEMBL genome browser 94) using the aligner TopHat v.2.0.13 [8]. Gene expression estimation was performed using Cufflinks v.2.2.1 [9]. To normalize sequencing depth among samples, FPKM values were calculated.

### Data preprocessing and gene selection

**Fig. 1:**
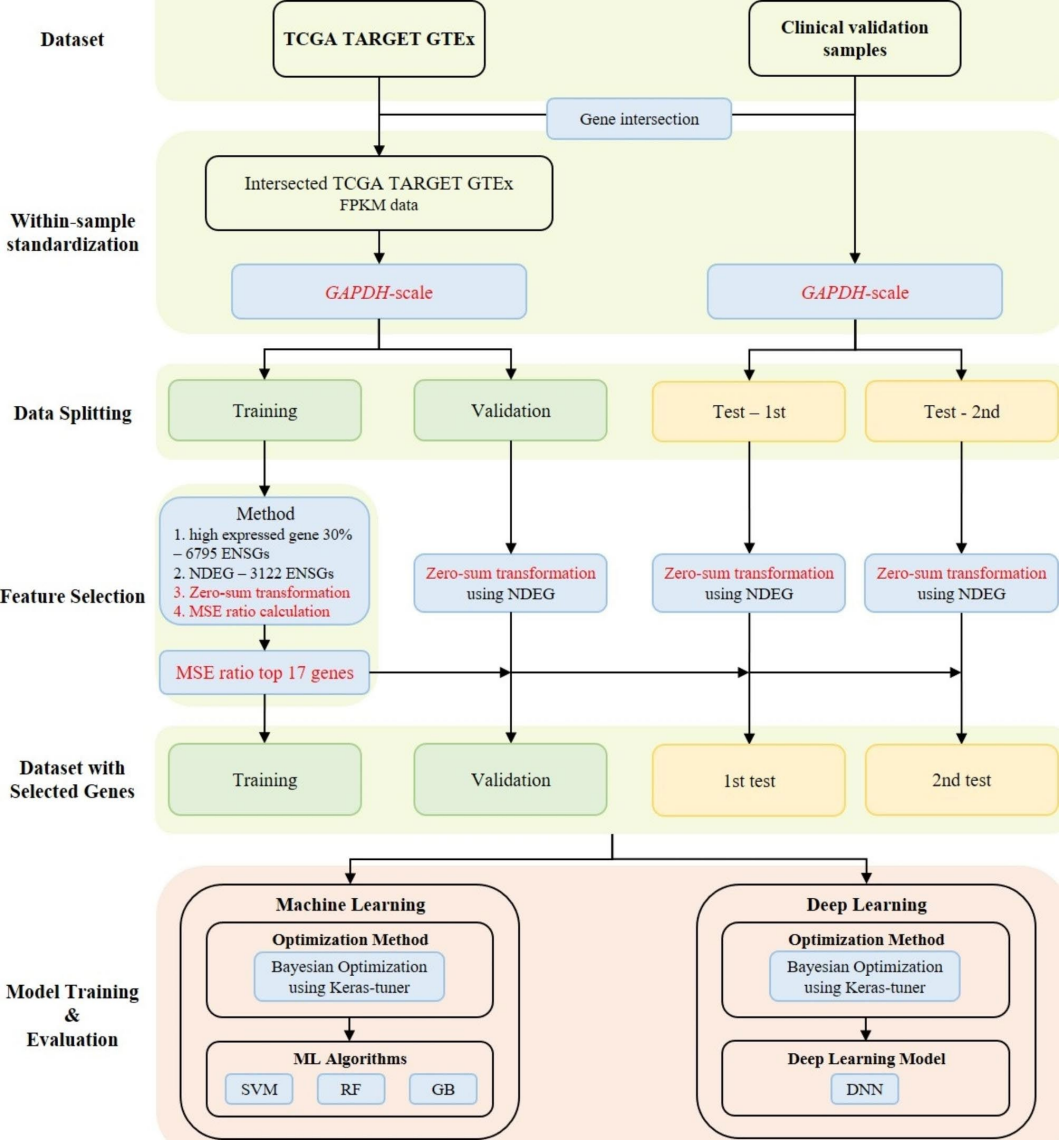
An overall workflow of the proposed classifiers approach. Two datasets, TCGA TARGET GTEx and test set, were used in this study. After standardizing the two datasets, they were split for training, validation, and testing. Features were selected only from the training set and were used to build classifiers. To obtain classifiers with the highest performance, we employed Bayesian optimization for hyperparameter tuning. TCGA = The Cancer Genome Atlas; TARGET = tumor alterations relevant for genomics-driven therapy; GTEx = Genotype-Tissue Expression; FPKM = fragment per kilobase of transcript per million mapped reads; ENSGs = Ensembl genes; NDEG = not differentially expressed genes; MSS = mean sum of squares; ML = machine learning; DNN = deep feedforward neural network; SVM = support vector machine; RF = random forest; GB = gradient boosting

Data preprocessing consisted of three steps: within-sample standardization, data splitting, and feature selection. The preprocessing steps are depicted in Fig. 1. To reduce the scale difference between samples, within-sample standardization was performed using the *GAPDH* gene, also known as glyceraldehyde-3-phosphate dehydrogenase, as a reference. *GAPDH* is a widely used housekeeping gene that is consistently expressed in cells, maintaining relatively stable expression levels regardless of cellular conditions. It is commonly employed as a reference gene for normalizing gene expression data and comparing expression levels across various conditions. Briefly, we subtracted the transformed FPKM value of *GAPDH* (log2 transformed after adding 0.001) from that of each gene for each sample. The training/validation set were randomly split into a training (n = 117) and a validation set (n = 28) in a 4:1 ratio. Both sets consisted of normal uterine tissue and leiomyosarcoma samples in the same proportion. The training set comprised 92 normal samples and 25 leiomyosarcoma samples, while the validation set included 22 normal samples and 6 leiomyosarcoma samples (Table 1).

The number of genes shared by the training/validation and test sets was 22,651, of which we selected 6,795 genes with an average expression FPKM value of upper 30% in the training set. Under the hypothesis that non-malignant leiomyoma samples share similar gene expression profile to normal samples, we narrow down candidate genes. For this, we use leiomyoma samples in the test 1st set (n = 8), another leiomyoma samples in the 2nd data set left over and not used feature selection and model training. We selected 3,122 genes from the 6,795 genes showed no significant differential expression between the leiomyoma samples in the 1st test set and the normal samples in training set (cutoff of var.test p-value < 0.05 and t.test p-value < 0.05). We defined the 3,122 genes as not differentially expressed genes between normal and leiomyoma samples (NDEG). We introduced a new transformation technique that sets the average of each sample to zero by subtracting the average of the sample, referred to as “zero-sum transformation” in this study. The zero-sum transformation was performed using 3,122 genes. To select genes with greater variance in leiomyosarcoma compared to normal samples, we computed the mean sum of squares (MSS) for each gene. The MSS was calculated separately for normal and leiomyosarcoma samples using the following equation:

1 $$MSS of genei=\frac{{x}_{1i}^{2}+{x}_{2i}^{2}+\dots +{x}_{ni}^{2}}{n}$$

where gene_*i*_ is *i*-th gene, with *i* = 1, 2, …, k, k is the number of genes, n is the number of samples, and x_n*i*_ is the zero-sum transformed FPKM value of *i*-th gene in the sample. Then, MSS ratio was calculated by dividing the MSS of leiomyosarcoma by the MSS of normal.

2 $$MSS ratio of {gene}_{i}=\frac{{MSS}_{LMS} of {gene}_{i}}{{MSS}_{normal} of {gene}_{i}}$$

In the Eq. (2), MSS_LMS_ is MSS of uterine leiomyosarcoma and MSS_normal_ is MSS of normal uterine tissue.

Based on the point where the gap observed in the MSS ratio graph of the top 20 genes, we selected the top 17 genes with high MSS ratio (Supplementary Fig. 1). Like the training set, zero-sum transformation was performed using NDEG on the validation and test sets. The selected 17 genes from the training set were used to train, validate, and evaluate the performance of classifiers.

### Deep learning and machine learning algorithms

We evaluated several classifiers, including deep feedforward neural network (DNN), support vector machine (SVM), random forest (RF), and gradient boosting (GB), for our analysis. The DNN classifier utilized a hidden layer structure with a rectified linear unit (ReLU) activation function, and the output layer employed a sigmoid activation function. Batch normalization was implemented to address the vanishing gradient problem. Adam optimizer with a learning rate of 0.001 was selected, and binary crossentropy served as the loss function. The DNN classifier provided probability predictions for uterine leiomyosarcoma, and the samples were classified using a cutoff value of 0.5. For the SVM classifier, we explored different parameters including kernel type, C, and gamma. The RF classifier was assessed with variations in parameters such as the number of estimators, maximum depth, and maximum features. The GB classifier involved tuning the learning rate, number of estimators, maximum depth, and maximum features. We utilized the SVM, RandomForestClassifier, and GradientBoostingClassifier algorithms provided by the scikit-learn library for implementing the respective classifiers.

### Hyperparameter tuning and evaluation

The DNN classifier had five hyperparameters including dropout rate, the number of hidden layers, the number of hidden layer nodes, batch size, and epochs. To determine the optimal set of hyperparameters, we performed Bayesian optimization of DNN classifier provided in the Keras-Tuner library [10]. We defined binary crossentropy as an objective function for optimization and the total number of trials was set at 500. We considered configurations of hyperparameters composed of dropout rate from 0.1 to 0.7, the number of hidden layers from 2 to 8, the number of hidden layer nodes from 2 to 32, batch size from 8 to 64, and epochs from 30 to 200 for the DNN classifier. Binary crossentropy was used as a metric function to select the classifier with the best performance. The structure of the selected classifier is depicted in Supplementary Fig. 2. The hyperparameter optimization of SVM, RF, and GB classifiers was performed in the same manner, except for the choice of objective function, which was accuracy in all cases. The configurations of hyperparameters and the hyperparameter values with the best performance of each classifier are shown in Supplementary Table 1.

The performances of the classifiers were evaluated using metrics such as accuracy, sensitivity, specificity, balanced accuracy, and area under the curve (AUC). The accuracy and AUC were utilized to determine the best classifier.

### Functional analysis of genes

The selected genes were subjected to functional analysis using GeneMANIA (https://genemania.org/). Significant functions were determined with false discovery rate (FDR) < 0.1 as cutoff.

## Results

### Gene expression

**Fig. 2:**
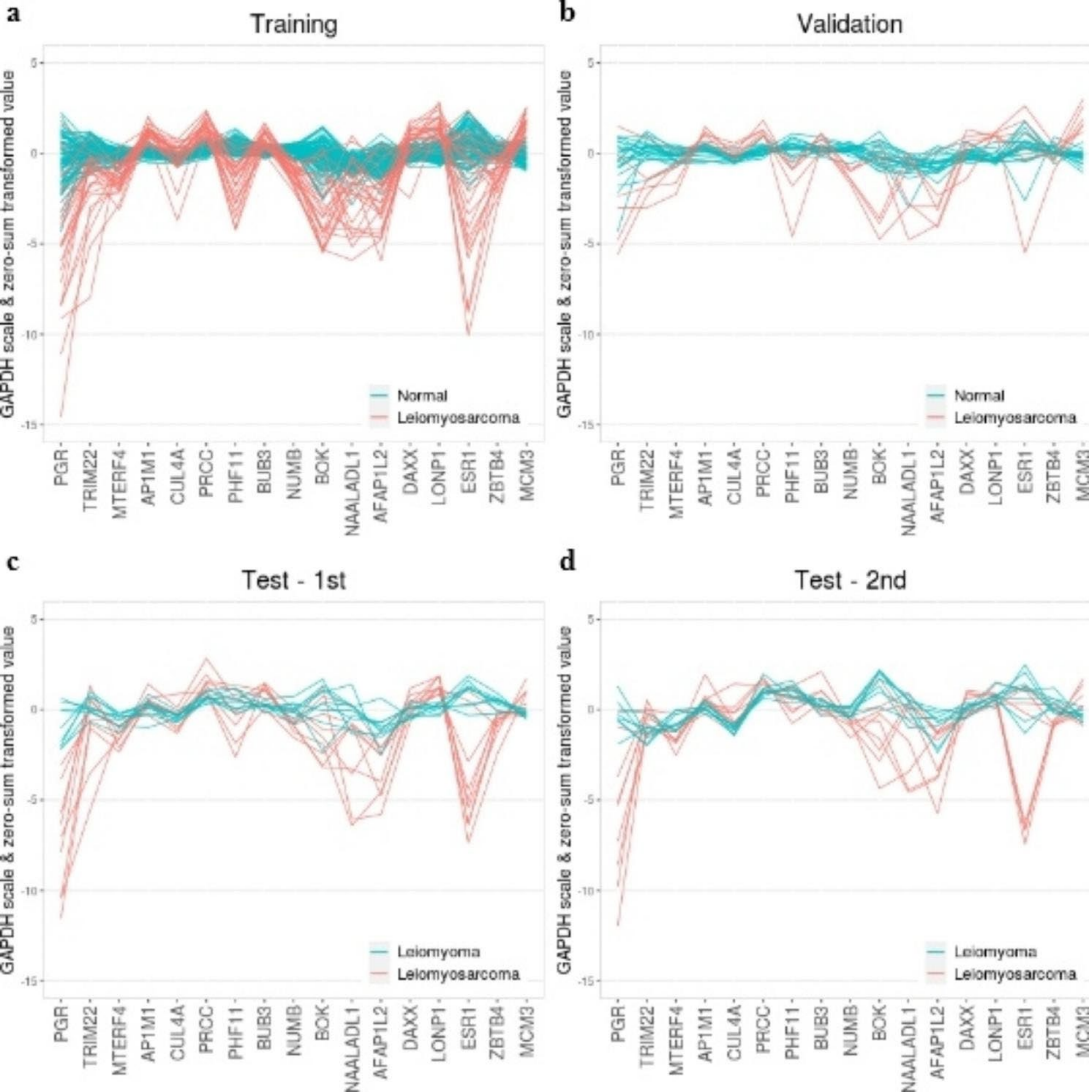
Expression patterns between datasets of 17 selected genes. The expression patterns of selected 17 genes in training set (a), validation set (b), 1st test set (c), and 2nd test set (d). Gene expression patterns in the normal (n = 114 samples from uterus, cervix, cervix uteri, endometrium) and leiomyoma groups (n = 17 samples) are stable, while they are dynamic in the leiomyosarcoma groups (n = 49 samples). The patterns were similar in all datasets. Color corresponds to the label. The y-axis represents *GAPDH*-scaled and zero-sum transformed expression values. These values were used to train the classifiers

**Fig. 3:**
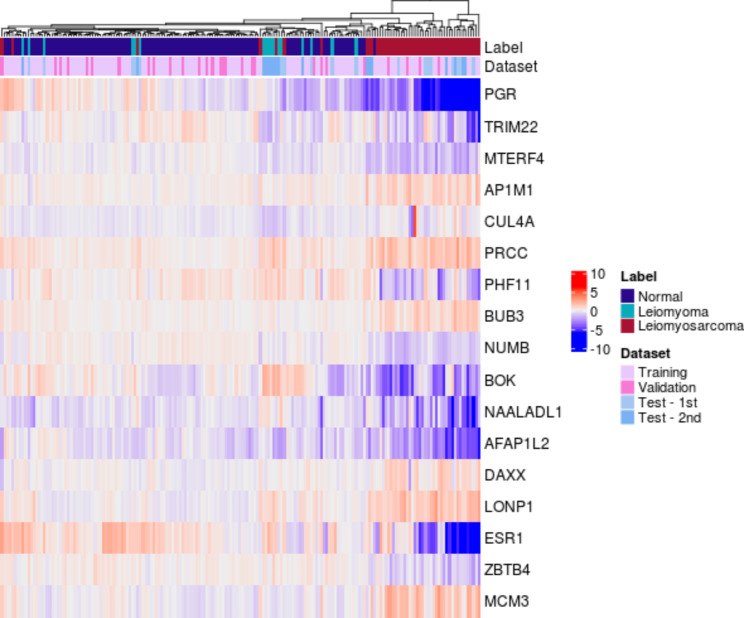
Graphical representation of the selected 17 genes. Heat map plot of the differentially expressed 17 genes in normal uterine tissue and leiomyosarcoma. Normal (n = 114 samples) and leiomyoma (n = 17 samples) show similar gene expression profiles, whereas leiomyosarcoma (n = 49 samples) displays a different profile. The distinct cluster of leiomyosarcoma can be seen in Label annotation. In the Dataset annotation, clusters were not identified. The scaled expression levels of the upregulated genes and downregulated genes are exhibited as red and blue, respectively

In the training set, normal uterine tissue samples showed consistently low MSS values for the selected genes, indicating conserved gene expression levels (Supplementary Table 2). Conversely, uterine leiomyosarcoma samples exhibited high MSS values. Specifically, genes such as *PGR*, *TRIM22*, *BOK*, *NAALADL1*, *AFAP1L2*, and *ESR1* showed MSS values greater than 5 in uterine leiomyosarcoma samples. This pattern was not only observed in the training set but also in the validation and test sets, where the expression levels of the selected genes in uterine leiomyosarcoma samples displayed greater variability compared to those in normal uterine tissue and uterine leiomyoma samples (Fig. 2). Thus, leiomyosarcoma is expected to have different expression pattern when it is compared to non-cancer samples. The visualized clustering pattern clearly demonstrates the distinction, as the uterine leiomyosarcoma samples are clustered separately from the normal uterine and uterine leiomyoma samples (Fig. 3). A total of 17 genes included in the classifiers are listed with their MSS ratio in Supplementary Table 2. Functional analysis showed that the selected genes were mainly associated with DNA replication preinitiation complex, DNA strand elongation, and protein-DNA complex (Supplementary Table 3).

### Performance of classifiers

**Fig. 4:**
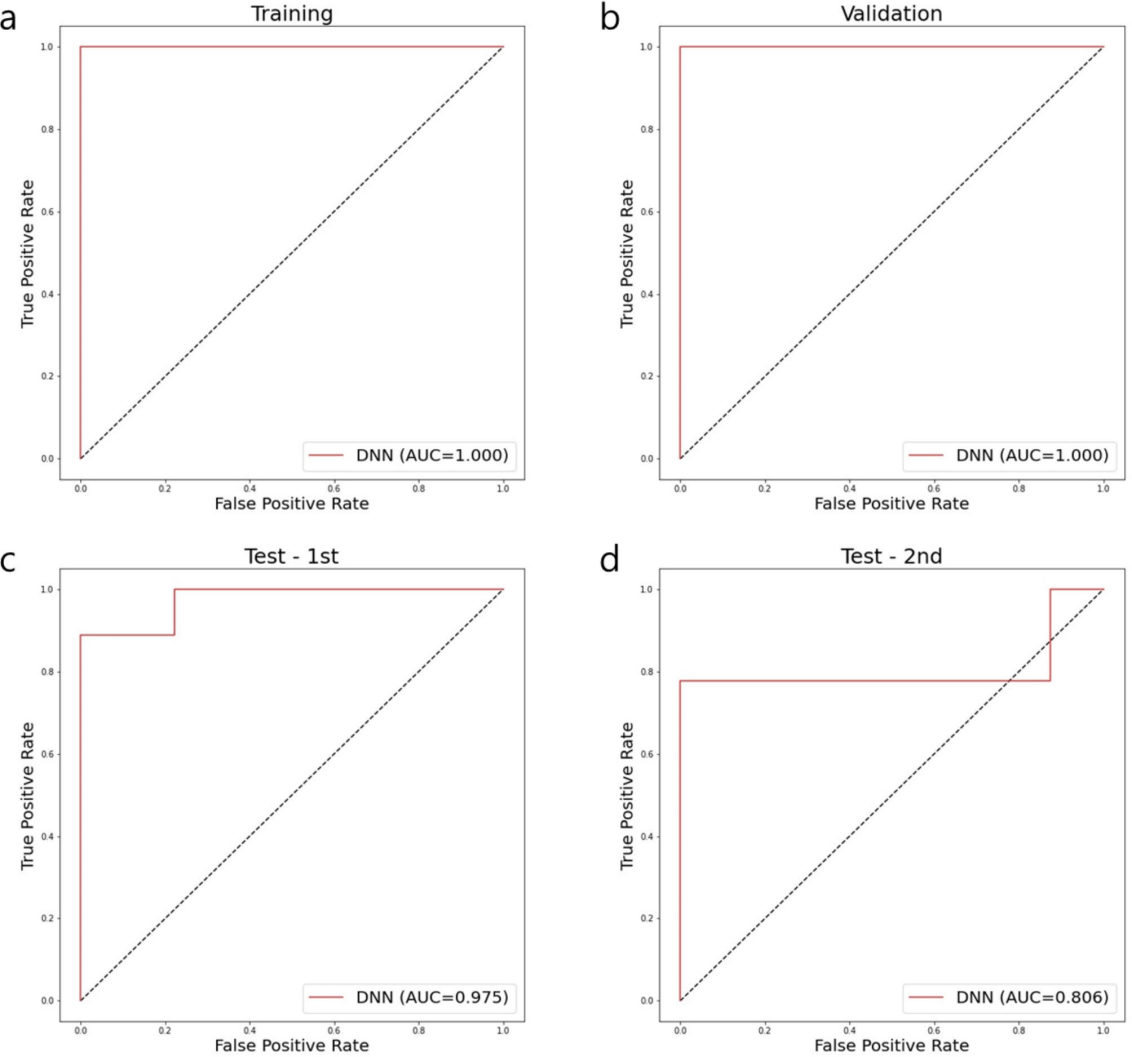
Prediction performance (AUC) of tuned DNN classifier in train, validation, 1st, and 2nd test. Receiver operating characteristic (ROC) curves of the classifier in training set **(a)**, validation set **(b)**, 1st test set **(c)**, and 2nd test set **(d)**. The area under the curve (AUC) values are shown in the plot legend

**Fig. 5:**
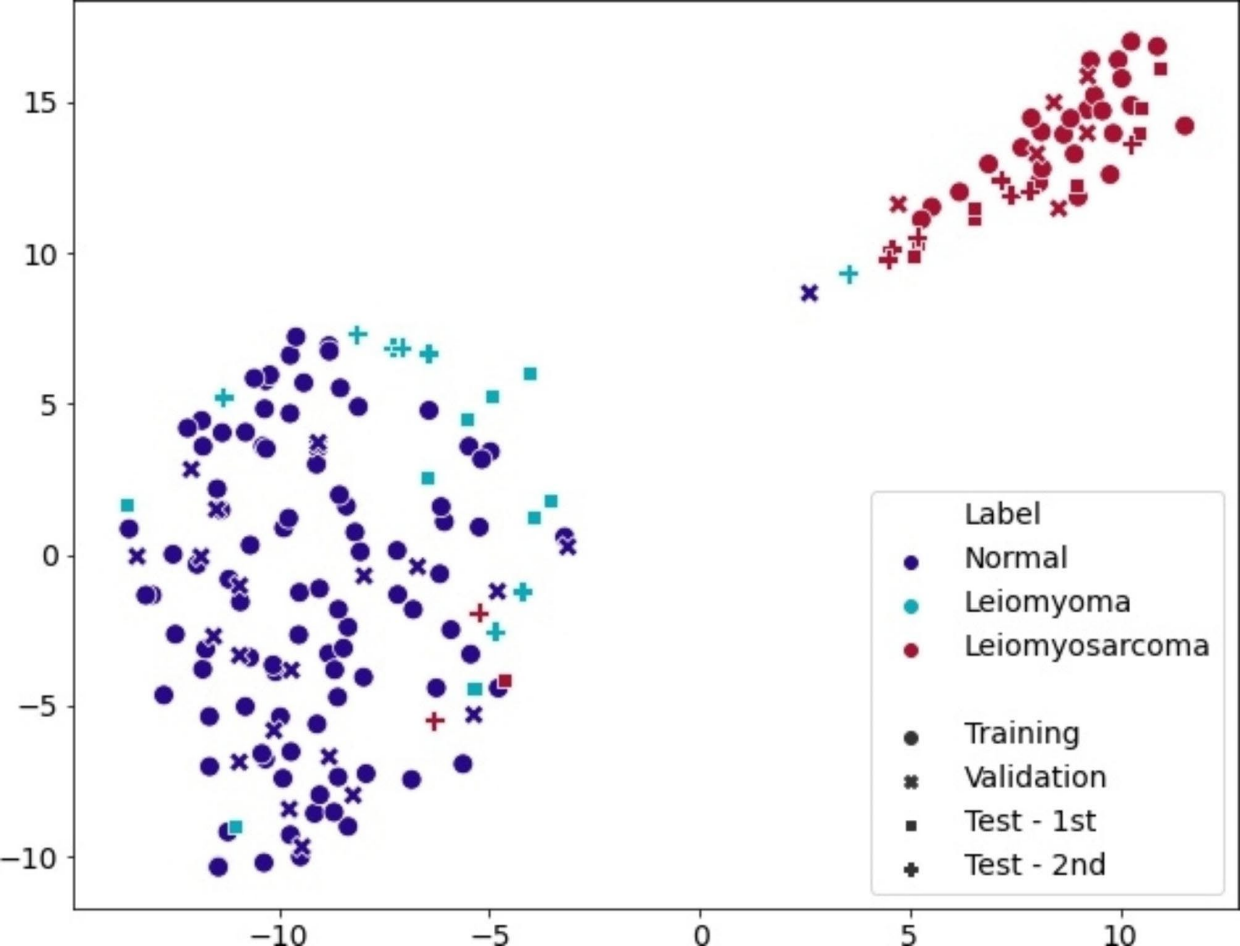
Visualization of the last hidden layer of DNN classifier. The t-SNE plot of the last hidden layer of the DNN classifier. Normal and leiomyoma samples were clustered, and leiomyosarcoma samples formed different clusters. Distinct clusters between the normal and leiomyoma group and leiomyosarcoma group were identified

Machine learning methods, including SVM, RF, GB, and DNN, were trained using normal uterine tissue and leiomyosarcoma samples, and they showed AUC values of 1.000 in both training and validation sets. We evaluated the performance of classifiers using newly collected clinical samples consisting of uterine leiomyoma and leiomyosarcoma (1st and 2nd test set in the figures). The AUC values of SVM, RF, GB, and DNN classifiers from the 1st test set were 0.926, 0.938, 0.975, and 0.975 respectively and those from the 2nd test set were 0.792, 0.792, 0.819, and 0.806. In comparison among SVM, RF, GB, and DNN classifiers, the DNN classifier had the highest accuracy and AUC value in the 1st test set (Fig. 4, Supplementary Fig. 3). The probability of uterine leiomyosarcoma for the test set using the DNN classifier is depicted in Supplementary Fig. 4. Accuracy, sensitivity, specificity, and balanced accuracy of DNN classifier in the 1st test set were 0.944, 0.889, 1.000, and 0.944, respectively; those in the 2nd test set, which is an independent validation set, were 0.882, 0.778, 1.000, and 0.889, respectively (Table 2). Those of SVM, RF, and GB classifiers for 1st and 2nd test sets are summarized in Supplementary Table 4. On visualization of the last hidden layer of the DNN classifier, the last hidden layer representations of uterine leiomyoma were closer to those of normal uterine tissue than malignant tumor (Fig. 5).

**Table 2:** Confusion matrix in test sets

1st test set				
		Actual		
		Leiomyoma	Leiomyosarcoma	Subtotal
Prediction	Leiomyoma	9	1	10
	Leiomyosarcoma	0	8	8
	Subtotal	9	9	18
2nd test set				
		Actual		
		Leiomyoma	Leiomyosarcoma	Subtotal
Prediction	Leiomyoma	8	2	10
	Leiomyosarcoma	0	7	7
	Subtotal	8	9	17

## Discussion

### Previous studies and novelty of this study

To distinguish uterine leiomyosarcoma from leiomyoma, many studies have used imaging modalities, but only a few studies have used RNA or DNA profiles. A study including 13 leiomyoma and 13 leiomyosarcoma formalin-fixed paraffin-embedded (FFPE) tissue samples reported that tumor mutation was more frequent in leiomyosarcoma than in leiomyoma [11]. Another study including 20 leiomyoma and 10 leiomyosarcoma fresh/FFPE tissue samples found several genes differentially expressed between uterine leiomyoma and leiomyosarcoma [12]. One more study including 7 leiomyoma and 9 leiomyosarcoma tissue samples reported that the gene expression profile is different between uterine leiomyoma and leiomyosarcoma [13]. However, these studies did not attempt to build a classifier and did not report how accurately they can predict whether a tumor is leiomyoma or leiomyosarcoma.

To the best of our knowledge, the present study is the first to generate a transcriptome-based classifier and reported the performance of the classifiers in differential diagnosis of uterine leiomyoma and leiomyosarcoma. We showed that uterine leiomyoma and leiomyosarcoma can be distinguished using transcriptome analysis with good accuracy.

### Genes with high variability in uterine leiomyosarcoma

We identified genes that exhibited larger variance in uterine leiomyosarcoma compared to normal uterine tissue and uterine leiomyoma samples. Among these genes, the *PGR* gene stood out with the highest MSS ratio of 22.655. It encodes progesterone receptor (PGR), which plays a crucial role in regulating uterine function through antagonistic and synergistic interactions with estrogen receptors (ER) [14]. Additionally, *ESR1*, which encodes ER, ranked second in terms of MSS value in uterine leiomyosarcoma samples following *PGR*. *ESR1* is associated with gynecologic cancers, including breast cancer, endometrial cancer, and ovarian cancer [15–17]. Both PGR and ER have also been reported as positive prognostic markers for uterine leiomyosarcoma [18]. *TRIM22* displayed the second highest MSS ratio among the identified genes. Saito-Kanatani et al. reported *TRIM22* as a progesterone-responsive gene in Ishikawa endometrial cancer cells, suggesting its involvement in regulating progesterone actions in uterine cells [19].

In addition, *BOK*, *NAALADL1*, and *AFAP1L2* were identified as genes with MSS values greater than 5 in uterine leiomyosarcoma samples. Although not specific to gynecologic cancers, there have been reports on these genes in relation to other cancer types. *BOK*, also known as *BCL2*-related ovarian killer, is involved in regulating the cell cycle and the pro-apoptotic pathway [20]. Several studies have investigated the role of *BOK* in cancer cells, with Carberry et al. suggesting that BOK protein may serve as a prognostic marker in colorectal cancer, and Zeilstra et al. reporting an association between *BOK* expression and intestinal adenomas [21, 22]. *NAALADL1* encodes NAALADaseL, which is studied as a biomarker in prostate cancer. A previous study has discovered that the expression of *NAALADL1* is upregulated in neuroendocrine prostate cancer [23]. *AFAP1L2* belongs to the actin filament-associated protein (AFAP) family, which is known affect tumor cell proliferation, invasion, epithelial-mesenchymal transition and participate in tumor progression. *AFAP1L2* has been reported to be associated with tumor progression or suppression in various cancers, including prostate cancer, non-small-cell lung cancer, breast cancer and carcinogen-induced skin tumorigenesis [24–27].

The selected genes utilized in the proposed classifier have been reported to exhibit expression changes in various types of cancers. Particularly, they include genes associated with female hormones, which likely contributed to the predictive performance of the classifier in distinguishing uterine leiomyosarcoma from normal uterine tissue and uterine leiomyoma.

### Functional analysis

We conducted a functional enrichment test on the selected genes to discover the functions associated with uterine leiomyosarcoma. We have identified three terms related to DNA replication: “DNA replication preinitiation complex” (FDR = 4.3e-5), “DNA strand elongation” (FDR = 1.3e-3), and “protein-DNA complex” (FDR = 4.8e-3). Notably, a previous study reported an association between “DNA strand elongation” and uterine leiomyosarcoma [28]. *MCM3*, which is involved in these functions, belongs to the minichromosome maintenance (MCM) family and has been previously reported in relation to cancer [29]. MCM family consists of *MCM2* to *7*, and they play crucial roles in “cell cycle progression” and “regulating the initiation and progression of DNA replication” [30]. Especially, the expression of *MCM2* is significantly upregulated in uterine leiomyosarcoma compared to normal myometrium and uterine leiomyoma [13, 30, 31]. In this study, we observed a similar pattern in the expression of *MCM3*, one of the genes used in the proposed classifier. Hence, *MCM3* holds potential as a novel marker for uterine leiomyosarcoma.

### Use of normal uterine tissue instead of uterine leiomyoma in classifier training

In the training/validation set, we used normal uterine tissue instead of uterine leiomyoma due to the unavailability of public transcriptome data for leiomyoma. To address this limitation, during the feature filtering process, we excluded genes that showed differential expression between normal tissue and leiomyoma using the 1st test data. Despite being trained with normal uterine tissue and leiomyosarcoma samples, the proposed DNN classifier effectively distinguished leiomyosarcoma from leiomyoma in the test sets through the feature filtering process, demonstrating its accurate performance. We identified that normal uterine tissue and uterine leiomyoma had similar expression profiles in several visualizations. Moreover, genes used in the classifiers do not overlap with genes that are differentially expressed between uterine leiomyoma and normal tissue (Supplementary Fig. 4) [32]. Therefore, we believe that the substitution of leiomyoma with normal uterine tissue did not significantly affect the performance of the classifiers. We may improve the performance of the classifier if we collect more uterine leiomyoma samples in future studies.

### Variance of expression

Differentially expressed genes (DEGs) via mean difference test have been widely used in transcriptome analysis. In this study, we used a novel approach that focused on divergence of gene expression pattern. There have already been many studies focusing on the analysis of gene expression variability. There is evidence that genes with high variability of expression are often associated with disease phenotypes [33, 34]. Notably, the variance of gene expression in cancer tissues is higher than that in normal tissues [35–37]. We applied this insight that gene expressions in cancer tissues are heterogenous while their expressions in normal tissues are conserved, for distinguishing uterine leiomyosarcoma from normal uterine tissue and leiomyoma. There are several studies using variance of gene expression for gene selection. Roberts et al. classified cancer subtypes using variance-based gene selection [38]. Dinalankara et al. used the variance-ratio statistic to distinguish benign growths from cancerous growths [36]. Moreover, they observed that not only was the variability of gene expression higher in both adenoma and cancer samples than in normal samples, but also that the cancer samples had higher variability compared to adenomas. This characteristic was applied to the present study, and the classifiers successfully distinguished uterine leiomyosarcoma from leiomyoma in model evaluation. As far as we know, this is the first study classifying uterine leiomyoma and leiomyosarcoma using variance-based gene selection. We believe that this novel approach may be more practically applied to the differential diagnosis between cancer and normal, and even disease and normal states.

### Impact of this study

The impact of preoperative differential diagnosis between uterine leiomyoma and leiomyosarcoma is huge. Unnecessary surgery can be avoided in women with uterine leiomyoma and early surgery can be performed in women with uterine leiomyosarcoma. Additionally, an appropriate surgical approach such as laparotomy instead of laparoscopy can be used in women with uterine leiomyosarcoma.

### Limitations

This study has several limitations. Firstly, due to the unavailability of transcriptome data for leiomyoma, we used normal uterine tissue as a substitute in the training/validation set. Secondly, the sample size in the training, validation, and test sets was relatively small. However, compared to previous studies [11–13], the current study can be considered relatively large in scale. Thirdly, the underlying reasons for the differential expression of the selected genes between uterine leiomyoma and leiomyosarcoma were not investigated in this study, and further research is needed to address this aspect.

## Conclusion

We successfully developed a transcriptome-based classifier using publicly available transcriptome data and showed that the classifier accurately distinguished uterine leiomyosarcoma from leiomyoma. The differential diagnosis via transcriptome analysis of preoperative biopsy specimens may lead to optimization of treatment plans in women with uterine leiomyoma or leiomyosarcoma.

### Electronic supplementary material

Below is the link to the electronic supplementary material.

Supplementary Material 1

## Data Availability

and materials. The RNA-Seq data from this publication have been deposited to the NCBI GEO repository (https://www.ncbi.nlm.nih.gov/geo) and can be accessed with the dataset identifier GSE222045.

## Code Availability

The code used for preprocessing and classification is available on GitHub (https://github.com/ForetellMyHealth/Diane.git).
