# Supplementary material for: A differential diagnosis between uterine leiomyoma and leiomyosarcoma using transcriptome analysis
Source: BMC Cancer. 2023 Dec 8;23:1215. doi: 10.1186/s12885-023-11394-0 (PMC10709939; doi:10.1186/s12885-023-11394-0)
Supplement: Supplementary file 1 — Supplementary Material 1 [file 12885_2023_11394_MOESM1_ESM.docx]

**Supporting information**

**Supplementary Figure 1. Structure of the best classifier DNN after hyperparameter tuning.**


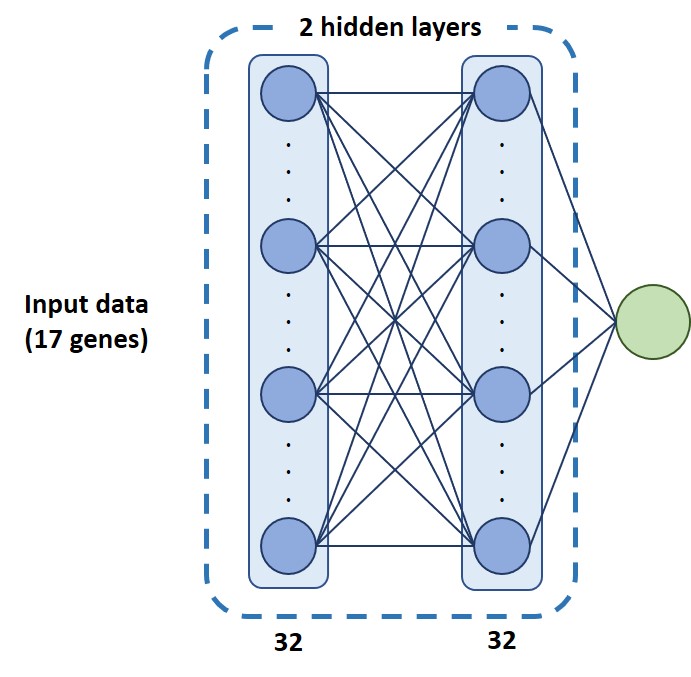


DNN classifier takes selected 17 genes as input. We obtained optimal hyperparameter set through Bayesian optimization. The classifier consists of two hidden layers with ReLU activation function and each hidden layer has 32 nodes. The last layer with a single node for output uses sigmoid. The output represents the probability of uterine leiomyosarcoma. *Abbreviations*: DNN = deep feed-forward neural network; ReLU = rectified linear unit.

**Supplementary Figure 2. Performance comparison of the tuned four classifiers (DNN, SVM, RF, and GB) in training, validation, 1st, and 2nd test sets.**


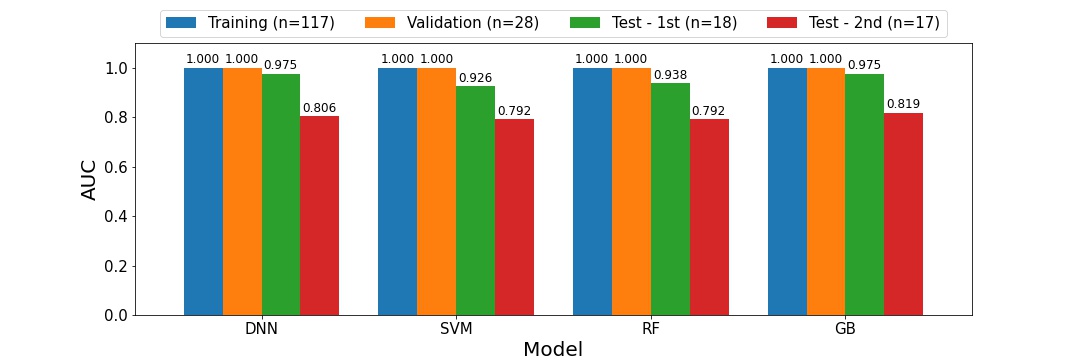
Each bar represents the AUC values of classification. Colors correspond to the datasets. The numbers of samples are shown in the plot legend. *Abbreviations*: DNN, deep feed-forward neural network; SVM, support vector machine; RF, random forest; GB, gradient boosting; AUC, area under the curve.

**Supplementary Figure 3. Prediction plot of probability of uterine leiomyosarcoma for the test set.**
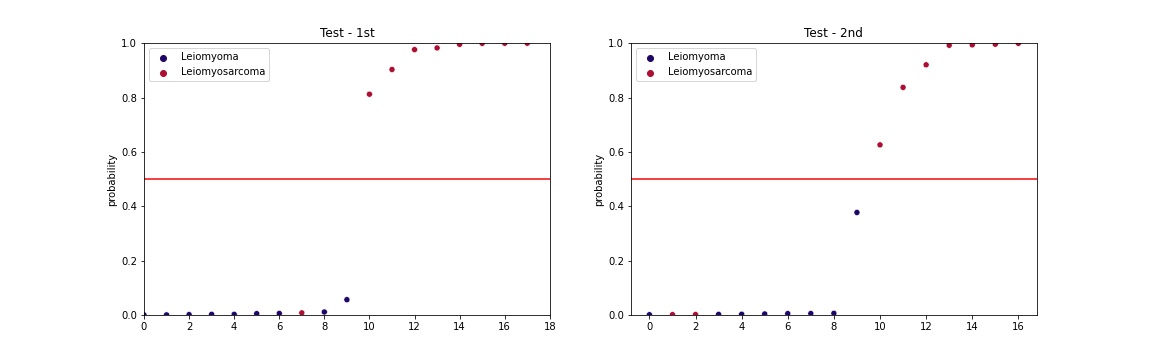


DNN classifier predicted the probability of uterine leiomyosarcoma. The samples were classified with cutoff values of 0.5. The x-axis and y-axis represent samples sorted by probability and predicted probability of uterine leiomyosarcoma, respectively. Samples are labeled with blue, if uterine leiomyoma and red, if uterine leiomyosarcoma. *Abbreviations*: DNN, deep feed-forward neural network.

**Supplementary Figure 4. Identification of overlap between differentially expressed genes of normal vs. leiomyoma and normal vs. leiomyosarcoma.**


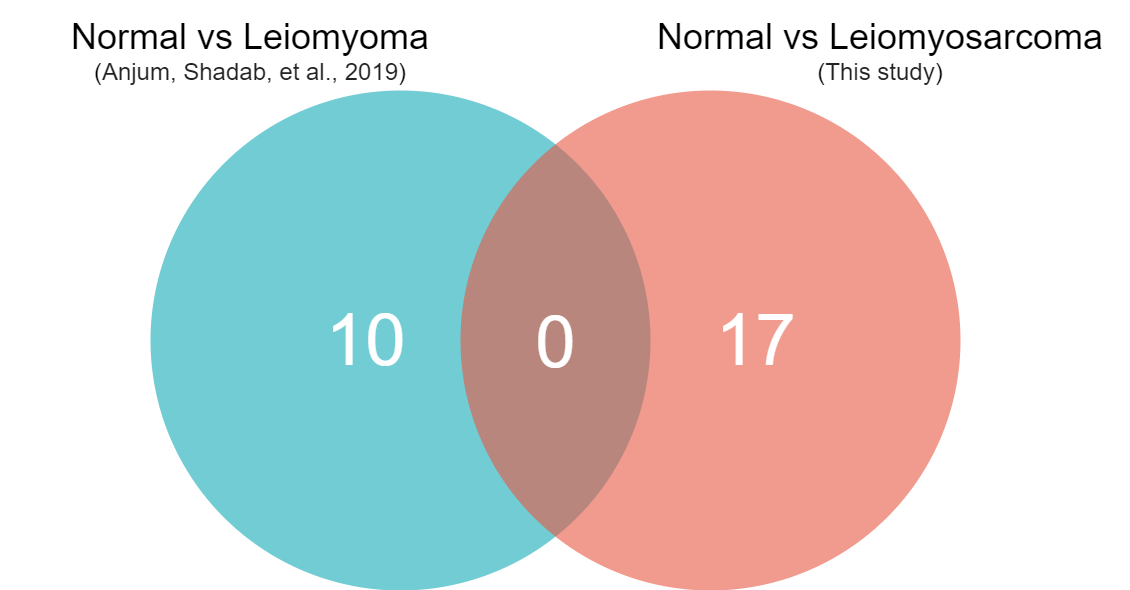
Venn diagram represents the differentially expressed gene number of normal vs. leiomyoma and normal vs. leiomyosarcoma. Genes in normal vs. leiomyosarcoma are selected genes from the proposed DNN classifier. Genes in normal vs. leiomyoma are selected genes reported by Anjum, et al. [18]. These genes do not overlap with the genes used in the classifier. *Abbreviations*: DNN, deep feed-forward neural network.

**Supplementary Table 1. Hyperparameter configurations selected via the Keras-Tuner library.**

| **Classifier** | **Hyperparameter** | **Possible values** | **Best value** |
| --- | --- | --- | --- |
| DNN | Dropout rate | 0.1 to 0.7 | 0.1 |
|  | The number of hidden layers | 2 to 8 | 2 |
|  | The number of hidden layer Nodes | 2 to 32 | [32, 32] |
|  | Batch size | 8 to 64 | 25 |
|  | Epochs | 30 to 200 | 200 |
| SVM | Kernel | linear, poly, rbf, sigmoid | linear |
|  | C | 0.01, 0.1, 1, 10, 100 | 100 |
|  | Gamma | 0.001, 0.01, 0.1, 0.5, 1, 10 | 10 |
| RF | n_estimators | 100, 200, 300, 400, 500 | 200 |
|  | max_depth | 5 to 30 | 17 |
|  | max_features | 3 to 17 | 15 |
| GB | Learning rate | 0.0001 to 0.5 | 0.3357 |
|  | n_estimators | 100, 200, 300, 400, 500 | 100 |
|  | max_depth | 5 to 30 | 25 |
|  | max_features | 3 to 17 | 3 |

**Supplementary Table 2. List of genes used in the classifiers and values of the calculation method used for feature selection.**

| **Gene** | **Gene symbol** | **Leiomyosarcoma MSS**  **(A)** | **Normal MSS**  **(B)** | **MSS Ratio**  **(A / B)** |
| --- | --- | --- | --- | --- |
| ENSG00000082175 | PGR | 37.663 | 1.662 | 22.655 |
| ENSG00000132274 | TRIM22 | 6.130 | 0.284 | 21.583 |
| ENSG00000122085 | MTERF4 | 2.508 | 0.121 | 20.799 |
| ENSG00000072958 | AP1M1 | 1.464 | 0.073 | 19.979 |
| ENSG00000139842 | CUL4A | 2.043 | 0.111 | 18.482 |
| ENSG00000143294 | PRCC | 2.253 | 0.126 | 17.854 |
| ENSG00000136147 | PHF11 | 4.243 | 0.244 | 17.391 |
| ENSG00000154473 | BUB3 | 0.867 | 0.053 | 16.455 |
| ENSG00000133961 | NUMB | 1.396 | 0.090 | 15.443 |
| ENSG00000176720 | BOK | 12.562 | 0.821 | 15.292 |
| ENSG00000168060 | NAALADL1 | 8.427 | 0.554 | 15.202 |
| ENSG00000169129 | AFAP1L2 | 9.347 | 0.632 | 14.800 |
| ENSG00000204209 | DAXX | 1.223 | 0.083 | 14.753 |
| ENSG00000196365 | LONP1 | 2.489 | 0.170 | 14.655 |
| ENSG00000091831 | ESR1 | 16.827 | 1.159 | 14.520 |
| ENSG00000174282 | ZBTB4 | 1.694 | 0.122 | 13.834 |
| ENSG00000112118 | MCM3 | 2.770 | 0.202 | 13.747 |

*Abbreviations*: MSS, mean sum of squares.

**Supplementary Table 3. Functional analysis of the selected 17 genes.**

| **Function** | **FDR** |
| --- | --- |
| DNA replication preinitiation complex | 4.32.E-05 |
| DNA strand elongation | 1.27.E-03 |
| protein-DNA complex | 4.79.E-03 |
| pigment accumulation | 4.90.E-03 |
| pigment cell differentiation | 3.61.E-02 |
| developmental pigmentation | 5.44.E-02 |
| pigment granule organization | 5.44.E-02 |

*Abbreviations*: FDR, false discovery rate.

| Model | Test – 1st | | | | | Test – 2nd | | | | |
| --- | --- | --- | --- | --- | --- | --- | --- | --- | --- | --- |
|  | Accuracy | Sensitivity | Specificity | Balanced accuracy | AUC | Accuracy | Sensitivity | Specificity | Balanced accuracy | AUC |
| DNN | 0.944 | 0.889 | 1.000 | 0.944 | 0.975 | 0.882 | 0.778 | 1.000 | 0.889 | 0.806 |
| SVM | 0.833 | 0.667 | 1.000 | 0.833 | 0.926 | 0.588 | 0.222 | 1.000 | 0.611 | 0.792 |
| RF | 0.778 | 0.778 | 0.778 | 0.778 | 0.938 | 0.765 | 0.667 | 0.875 | 0.771 | 0.792 |
| GB | 0.833 | 0.778 | 0.889 | 0.833 | 0.975 | 0.706 | 0.667 | 0.75 | 0.708 | 0.819 |

**Supplementary Table 4. Prediction performance of classifiers on 1^st^ and 2^nd^ test sets.**

*Abbreviations*: AUC, area under the curve; DNN, deep feed-forward neural network; SVM, support vector machine; RF, random forest; GB, gradient boosting.
